# The mechano-sensing role of the unique SH3 insertion in plakin domains revealed by Molecular Dynamics simulations

**DOI:** 10.1038/s41598-017-11017-2

**Published:** 2017-09-15

**Authors:** Csaba Daday, Katra Kolšek, Frauke Gräter

**Affiliations:** 1 Interdisciplinary Center for Scientific Computing (IWR), Heidelberg University, Mathematikon, INF 205, 69120 Heidelberg Germany; 2 Heidelberg Institute for Theoretical Studies, Schloß-Wolfsbrunnenweg 35, 69118 Heidelberg, Germany

## Abstract

The plakin family of proteins, important actors in cross-linking force-bearing structures in the cell, contain a curious SH3 domain insertion in their chain of spectrin repeats (SRs). While SH3 domains are known to mediate protein-protein interactions, here, its canonical binding site is autoinhibited by the preceding SR. Under force, however, this SH3 domain could be released, and possibly launch a signaling cascade. We performed large-scale force-probe molecular dynamics simulations, across two orders of magnitude of loading rates, to test this hypothesis, on two prominent members of the plakin family: desmoplakin and plectin, obligate proteins at desmosomes and hemidesmosomes, respectively. Our simulations show that force unravels the SRs and abolishes the autoinhibition of the SH3 domain, an event well separated from the unfolding of this domain. The SH3 domain is free and fully functional for a significant portion of the unfolding trajectories. The rupture forces required for the two proteins significantly decrease when the SH3 domain is removed, which implies that the SH3 domain also stabilizes this junction. Our results persist across all simulations, and support a force-sensing as well as a stabilizing role of the unique SH3 insertion, putting forward this protein family as a new class of mechano-sensors.

## Introduction

Mechanotransduction is known to be an important biological process rendering tissues both resilient and responsive towards their mechanical environment. Enormous progress has been recently made in identifying some of the molecular players responsible for transducing and sensing mechanical forces. This is particularly true for focal adhesion sites, where the list of molecules with established mechano-sensing role is steadily growing, and includes integrin, talin, vinculin, p130Cas, focal adhesion kinase and others^1, 2^.

The hallmark of mechano-sensing is a functional change of a biomolecule when subjected to a mechanical force^3^. Typically, in case of cytosolic proteins, an enzymatic or binding site of a protein is inhibited in absence of a mechanical stimulus. This cryptic site becomes exposed under force, resulting in activation and downstream signaling events. We hypothesized that the plakin family, localized at various types of cellular junctions and critical for tissue integrity, serves as a new class of mechano-sensing proteins.

The plakin family of cytolinker proteins^4–6^ has long-established physiological relevance in several different biological tissues. Proteins in this family share a central plakin domain, consisting mainly of spectrin repeat domains. Spectrin repeats^7^ (SRs) are the main building blocks of structural proteins, consisting of three helices A, B, and C (A and C being parallel and antiparallel to B) and generally containing about 100–120 residues in total. Their overall sequence identity is quite low, even when considering repeats from the same protein^8, 9^.

**Figure 1:**
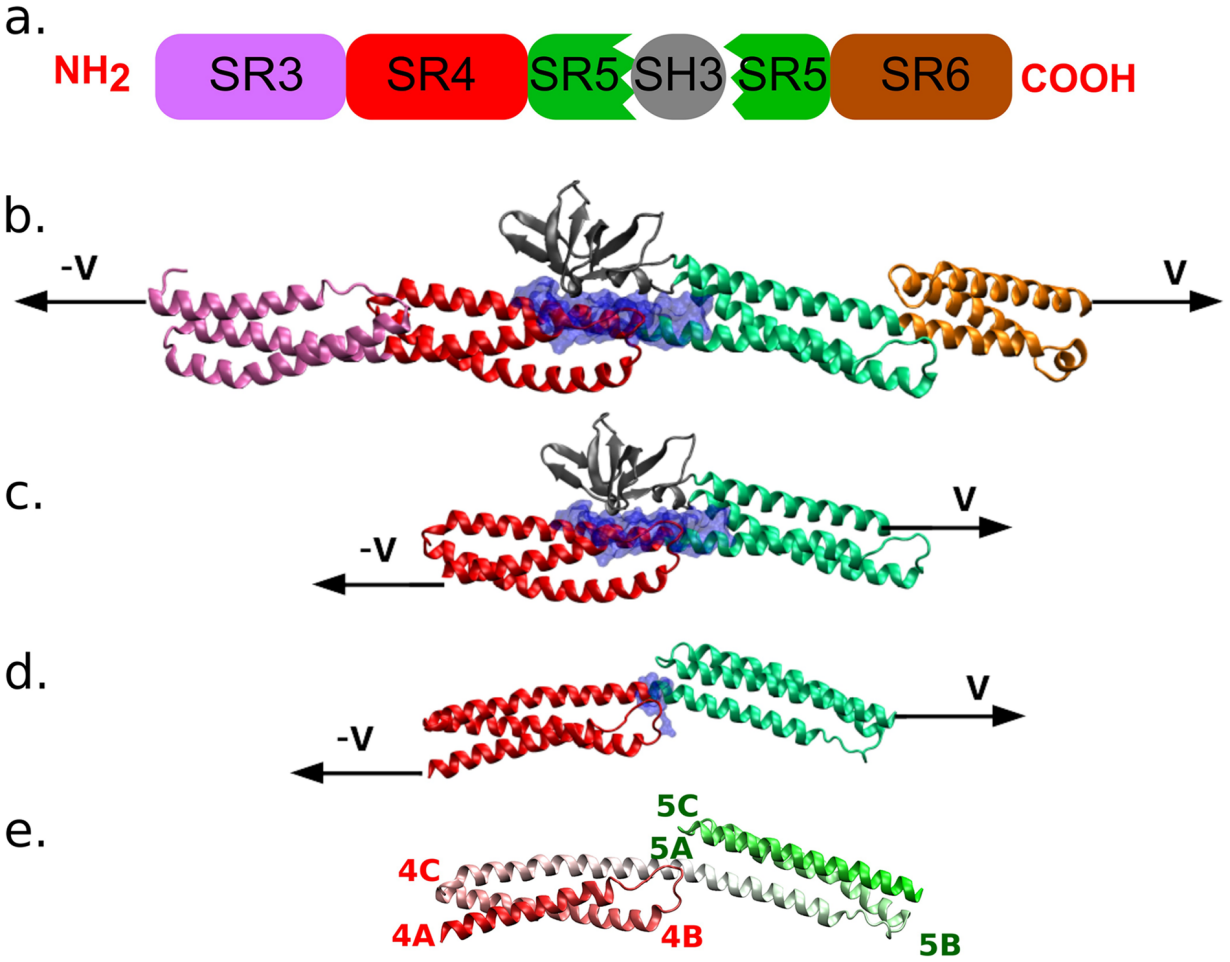
Plakin family proteins contain a curious SH3 domain insertion. (**a**) The three systems we study using force-probe molecular dynamics: (**b**) four spectrin repeats and an SH3 domain (only desmoplakin). (**c**) two spectrin repeats and an SH3 domain (desmoplakin and plectin), and (**d**) only two spectrin repeats with the SH3 domain removed (desmoplakin and plectin). The blue surface denotes the SH3-SR4 interface for cases. (**b**,**c)** and the SR4-SR5 interface for case. (**d)** shown are snapshots from MD simulations on desmoplakin, starting from PDB code 3R6N^18^. (**e**) The assignment of helices 4A-C and 5A-C in desmoplakin with the SH3 domain removed, for clarity. The structure is colored by residue index (N-terminal pure red, C-terminal pure green) and the labels of the helices are placed close to the N-terminus of all helices.

Remarkably, in all plakin family members as well as in a few spectrin family members, one of the spectrin repeats features a curious SH3 domain insertion (Fig. 1). SH3 domains are small $\beta$-barrel or $\beta$-sandwich domains. They are very commonly found in adaptor proteins, mediating protein-protein interactions by binding to partners. Sequence identity between various SH3 domains is relatively low considering their structural similarity^10^.

Since these two structural motifs, spectrin repeats and the SH3 domain, are very widespread, the unfolding of both SRs^11–15^ and individual SH3 domains^16, 17^ has been studied experimentally and computationally. However, the role of the SH3 insertion in spectrin repeats has remained largely elusive, especially as in this case, the canonical binding site of the SH3 is inhibited through interactions with the preceding spectrin repeat. The initial suggestion was that the purpose of the SH3 insertion is to stabilize the junction of the two adjacent SRs^18^, but the fact that SH3 domains are generally interacting with binding partners suggests an alternative (or additional) role, namely, that the SH3-SR interface can be opened and the SH3 domain thereby activated under force.

In fact, the hypothesis of force activation has some experimental support for at least two proteins featuring the unique SH3 insertion into spectrin repeats. For nonerythroid $\alpha$-spectrin, a binding partner for the SH3 domain has been experimentally observed^19^ in the integrin signaling complexes, and similarly, the Ic isoform of plectin has been shown to interact with the microtubuli-associated protein 1 (MAP1) through the SH3 domain^20^. While neither of these studies directly show that this process is force-induced, they provide evidence for that the SH3 domain is released at least part of the time and is capable of binding other proteins.

Here, we study the force response and putative mechano-sensing mechanisms of desmoplakin and plectin, two members in the plakin family. Both proteins have been structurally resolved and are known to have roles in tissue integrity: 25 different disease mutants of desmoplakin related to arrythmogenic right ventricular cardiomyopathy (ARVC) have recently been identified^21^, while the knockout of plectin has been shown to disturb adherens junctions (AJ) by abolishing the vimentin-actin crosslinks^22^.

Desmoplakin is an obligate linker protein in the desmosome, linking the inner dense plaque (at the inner side of the cellular membrane) to intermediate filaments^23^. Desmosomes are specific to epithelial and cardiac tissue. The central plakin domain of desmoplakin contains six spectrin repeat domains (called SR3 through SR8). The third spectrin repeat, SR5, contains the mysterious SH3 domain insertion that is inhibited by interacting with the previous spectrin repeat, SR4^18^.

Plectin is a cytolinker protein present at several kinds of cellular junctions: AJ, focal adhesions, and also hemidesmosomes^24^. Plectin also contains a plakin domain with a very similar structure, including the SH3 domain insertion^25^. The immediate proximity of this insertion, namely, the spectrin repeats SR4-SR5, has a high structural similarity to desmoplakin and both proteins have high sequence identity to other plakin family proteins (23–64%, see SI).

Given the role of the SH3 domain as an adaptor protein, we can reasonably infer that the plakin family members’ SH3 domain can generally serve as a signaling agent once released from the spectrin repeat. We here set out to study this possibility using simulations, in particular, force-probe molecular dynamics (FPMD). FPMD^26^, also known as steered MD^27^, has been used to study the individual unfolding of both SRs^11, 13, 14^ and SH3s^17^, often in conjunction with experiments. In the following, we will apply this method to different constructs of desmoplakin and plectin (Fig. 1) to answer the following questions: (i) do the domains unfold simultaneously or with well-delimited force peaks, (ii) would the absence of the SH3 domain significantly change the nature of the unfolding pathways or the rupture forces, (iii) is the SH3 domain activated under force or does it unfold while autoinhibited, and (iv) can we identify relevant differences between the two considered proteins? Our simulation data suggests that the SH3 domain both stabilizes the spectrin repeats against unfolding and is also force-activated for downstream signaling. While the two plakin family members studied by FPMD simulations share the major unfolding mechanism, we find desmoplakin to unfold at higher forces and in more distinct steps than plectin, shedding light on their mechanotransduction role in their different cell junction environments.

## Methods

We use GROMACS version 5.0^28^ with the Amber99SB-ildn* force field^29, 30^ with Joung’s modified ion parameters^31^ and TIP3P^32^ water molecules. All bonds are frozen in our simulations using the LINCS procedure^33^. We use the integration time step of 5 fs made possible by the use of virtual sites^34^ for all hydrogen atoms. Further force-probe simulations with a time step of 2 fs on the largest construct (SR3–6 of desmoplakin) are presented in the Supplementary Information. In short, the choice of a 5 fs time step does not affect the order of unfolding events but apparently increases the rupture forces by approximately 20%. As we here are interested in the unfolding mechanism and relative stabilities, we proceeded with 5 fs time steps.

For both the equilibrium and the force-probe simulations, we use a modified velocity-rescaling thermostat^35^ at 300 K with a time constant of 0.1 ps for both the protein atoms and the non-protein atoms. Isotropic pressure coupling is implemented according to the Parrinello-Rahman scheme^36^, with the compressibility $4.5\cdot {10}^{-5}$ bar^−1^ and a time constant of 0.1 ps.

We use the Particle Mesh Ewald (PME)^37^ technique to treat long-range electrostatic interactions, with a grid spacing of 0.16 nm and cubic interpolation. Neighbor lists with a cutoff of 1 nm with the Verlet cutoff are updated every 25 time steps (0.125 ps).

We start our desmoplakin simulations from the crystal structure 3R6N^18^, and those of plectin from the crystal structure 3PE0^25^. The more recent 5J1H^38^ crystal structure features slight differences in one of the spectrin repeats but lacks the SH3 domain. We also repeat a part of the plectin simulations using an alternative model based on the 3PE0 (for the SH3 domain) and the recently published 5J1H crystal structures (for the SRs) as templates, which we denote as “hybrid” model in the following. We solvate the two structures in water and proceed to add Na^+^ and Cl^−^ ions with a concentration of 0.15 M.

In our equilibrium simulations, we use a dodecahedron box with at least 3.0 nm between periodic replicas of the protein, resulting in a system containing about 640k (for the 4SR + SH3 simulations) or 200k atoms (for the other simulations). After solvation and the addition of ions, we first perform an energy minimization using the steepest-descent method with a step size of 1 pm, tolerance of 20 kJ/mol/nm, and a maximum of 500000 steps. Thereafter, we perform 500 ps of NVT and 500 ps of NPT equilibration, both with a harmonic restraint on the protein atoms of 1000 kJ/mol/nm^2^. After 300 ns of equilibrium simulation, we choose the top 10 clusters in a cluster analysis on backbone motion with a cutoff of 0.09 nm on 6001 frames (extracted every 50 ps) as initial frames for the FPMD simulations. The rupture forces from FPMD show no significant correlation with the time at which the initial frames have been extracted from the equilibrium simulations. For the “hybrid” model of plectin, we perform an equilibration of 100 ns and a further 900 ns of production run, on which we do the previously mentioned cluster analysis to obtain the starting frames for FPMD simulations. We perform additional simulations of a plectin mutant as described in the supplementary information.

### Force-probe MD

In all of our pulling simulations, we subject the atoms of the N- and C-terminal residues to a harmonic potential. The minimum of the harmonic potential is at the position of the center of mass of the residues at $t=0$ and each spring is moving at velocities $v=\pm \frac{1}{2}{v}_{{\rm{pull}}}$ to reduce protein-water friction. The force constant is $k=$ 500 ${\rm{kJ}}/(\mathrm{mol}\cdot {{\rm{nm}}}^{{\rm{2}}})$ ≈ 830 $\mathrm{pN}/\mathrm{nm}$ in all simulations.

After the equilibrium simulations, we extract the central frames of the clusters obtained from the equilibrium simulations, keeping only the protein atoms. We align the protein in such a way as to have the x-axis parallel with the vector connecting the centers of mass of these two pulling groups. We then re-solvate the protein in an oblong cuboid box where the x-axis is long enough to contain the unfolded polypeptide. We do this in two slightly different ways for the two proteins. For desmoplakin, we solvate the protein so the initial conformation is at least at a 5.0 nm distance from its periodic image, which results in a box size of about $100\times 10\times 10$ nm^3^ and about 800k atoms (for the 4 SR + SH3 simulations, this is $165\times 9\times 9$ nm^3^ and a total number of 1.2 M atoms). For plectin, we use a two-step procedure due to a high variance of orientations during unfolding (see discussion below). First, we solvate the conformations in a “small” box of $60\times 12\times 12$ nm^3^ and then, when the length of the partially unfolded protein approaches 60 nm, we re-solvate the protein into a box size of $100\times 8\times 8$ nm^3^. This involves a total number of atoms of about 700k and 500k, respectively. During the re-solvation, we ignore information on the velocities for the atoms. Any artefacts due to this procedure will not change the order of unfolding events or the size of rupture forces as these are determined in the first half of the FPMD simulations. Between any (re-)solvation and the start of the pulling simulations, we performed an energy minimization and NVT/NPT equilibration with the parameters described in the previous subsection. For both proteins and all unfolding simulations, these box sizes ensure that the protein is at least 1 nm away from its periodic image, which is the cutoff used for van der Waals interactions.

We choose the pulling velocities ${v}_{{\rm{pull}}}$ of $1$, $\mathrm{1/3}$, $\mathrm{1/10}$, $\mathrm{1/30}$, and $\mathrm{1/100}$ nm/ns (for reasons described below, we apply only the fastest two pulling velocities to the 4SR + SH3 system), and proceed to unfold until all secondary structure has been lost, with the exception of the slowest pulling velocity, in which case we observe only the first rupture event. We sample the pulling forces at every 50 ps and study the rupture forces by smoothing the average force profiles ($F=\frac{1}{2}({F}_{1}-{F}_{2})$, where ${F}_{\mathrm{1,2}}$ are the forces at either end, which have opposite signs and usually very similar absolute values) with a Gaussian width consistent with an elongation of 0.1 nm (i.e., 0.1, 0.3, 1, 3, or 10 ns), and recording only the highest force of the first peak (defined as the first 20 nm of elongation). The trends reported here remain consistent when repeating the same analysis with a window of 1 nm instead.

We compute contact areas between two regions A and B (where A and B can be helices, strands, or domains) using the solvent-accessible surface area (SASA):^39^ ${{\rm{CA}}}_{A\leftrightarrow B}=\frac{1}{2}({{\rm{SASA}}}_{A}+{{\rm{SASA}}}_{B}-{{\rm{SASA}}}_{A+B})$. We define unfolding events as follows: For spectrin repeats, we measure the contact area between the inner two helices (SR3B-C, SR4B-C, SR5A-B, and SR6A-B) and for inter-domains, we measure the contact area between them (ignoring two residues on either side of the analyzed fragments, ensuring that the contact area eventually drops to zero even when the sequences are directly consecutive). We define an abnormally low value for these contact areas as: ${{\rm{C}}{\rm{A}}}_{{\rm{l}}{\rm{o}}{\rm{w}}}=({\rm{Q}}1-1.5\cdot ({\rm{Q}}3-{\rm{Q}}1))$ with Q1, Q3 being the first and third quartiles in the equilibrium simulations, respectively. We defined unfolding events as beginning when the contact areas first drop below $\frac{1}{2}{{\rm{CA}}}_{{\rm{low}}}$ and ending when the contact area goes below $0.002$ nm^2^ (the numerical accuracy of our analysis methods). For visual clarity, when the unfolding events defined like this involve an elongation of less than 2 nm, we increase the boxes to this size.

We use the Bell model^40, 41^ to fit the rupture forces observed to the loading rate:

1 $${F}_{{\rm{r}}}=\frac{{k}_{B}T}{{x}_{b}}\,\mathrm{ln}(\frac{kv{x}_{b}}{{k}_{B}T\cdot {k}_{{\rm{o}}ff}})=A\,\mathrm{ln}(\frac{kv}{B}),$$

where we ${x}_{b}$, $k$, $v$, ${k}_{{\rm{off}}}$ refer to the distance to the transition state, the spring stiffness (kept constant in our case), the pulling velocity (for which we use four different values in our simulations, as mentioned), and the rupture rate of the unperturbed system, respectively. $A$ and $B$ are combinations of constants for ease of reading.

When comparing rupture forces between systems, we consider differences between systems significant if they pass the Z-test (the difference in means is at least 2 $\sqrt{{\varepsilon }_{1}^{2}+{\varepsilon }_{2}^{2}}$, where ${\varepsilon }_{\mathrm{1,2}}$ are standard errors on the mean) for all of the fastest three pulling velocities, where there are 10 values available.

## Results

### Force unlocks the SH3 domain

**Figure 2:**
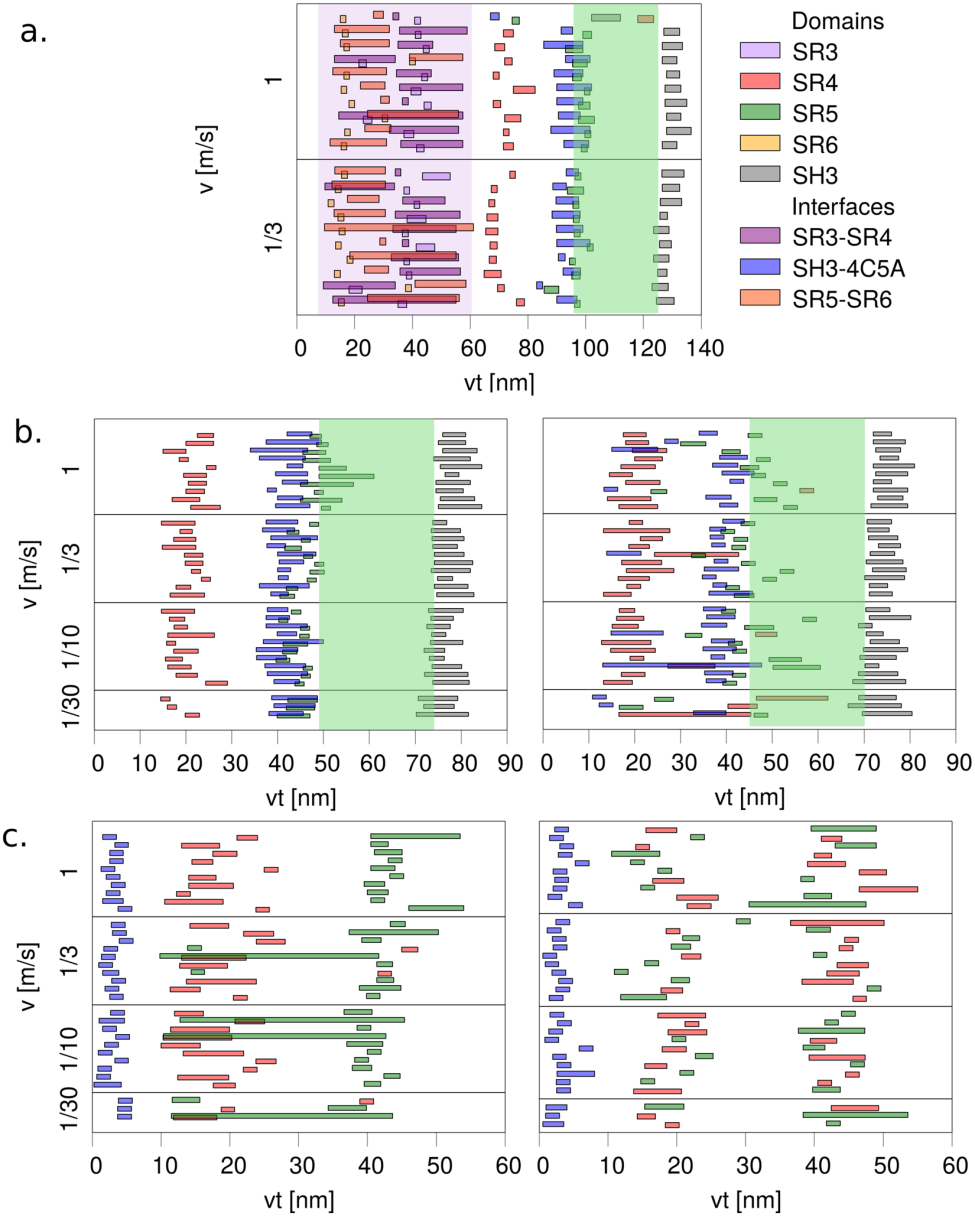
Unfolding events observed in all single force-probe simulations. Boxes extend from the start to the end of the loss in contact area, either within a single domain (SR3, SR4, SR5, SR6, and SH3) or at inter-domain interfaces (SR3-SR4, SH3-helix 4C5A, and SR5-SR6), see Methods for details. Each set of horizontally aligned boxes correspond to one FPMD trajectory. The unfolding events are represented as a function of inter-spring distance, which is very close to the net increase of the protein end-to-end distance. Three sets of systems are presented: (**a)** four spectrin repeats and an SH3 domain (only desmoplakin (20 runs)), (**b**) two spectrin repeats and an SH3 domain (left: desmoplakin and right: plectin (33 runs each)), and (**c**) only two spectrin repeats with the SH3 domain removed (left: desmoplakin and right: plectin (33 runs each)). The purple area in subplot a. shows that the domains SR3, SR6 and the associated interfaces are first to unfold (until vt $\,\approx \,60$ nm), while the green areas for subplots a, b show the minimum active area, i.e., the region of the unfolding trajectories after the SH3–4C5A interface is lost and before the beginning of the SH3 domain unfolding.

As a first set of simulations, we perform FPMD simulations on the largest available part of a plakin domain, namely of desmoplakin, which comprises four spectrin repeats, at two different pulling velocities. As shown in Fig. 2a, the outer two spectrin repeats (SR3, SR6) unfold first in all 20 pulling simulations. The SH3 domain always unfolds in the very last part of the trajectories, making its interaction with partners possible. However, the crucial SH3–4C5A interface remains intact while the outer two repeats unfold. Thus, the mechanical response of the central SRs close to the SH3 insertion and the putative SH3 activation clearly follow previous unfolding events in the plakin domain, and can be considered separately. With this in mind, in the following, we only study the force response of the central two spectrin repeats containing the SH3 domain: SR4 and SR5.

For desmoplakin, we observe (Fig. 2b, left) a very clear order of intra- and inter-domain unfolding events: First, SR4 unfolds at extensions of ~15–25 nm, then, the SH3-SR4 interface is severed and the SR5 unfolds at approximately the same time of extensions of ~40-60 nm. Between the moment in which the SH3 domain is released (~45–50 nm) and when it starts unfolding (~70–75 nm), the SH3 domain is free to bind to partners. We do not observe SH3 domain unfolding occuring before or during “activation” in any of the simulations. This would have been a distinct possibility, since helix 5 C often unfolds or loses contact in the very first unfolding event and thereby the SH3 domain is directly under stress.

The unfolding pathways of plectin (shown in Fig. 2b, right) are largely similar to those of desmoplakin. In particular, the unfolding of the SH3 domain still happens as the last event and, for at least an extension interval of 30 nm, it is intact and not autoinhibited. However, in 8 out of the 33 unfolding trajectories, it loses the interface with SR4 at even earlier moments in time, therefore being available for binding at an even earlier point. Further analysis on the residues with the interactions that last longest between the SH3 domain and the helix 4C5A reveals that this interface is lost in a very similar way in both proteins (see Supporting Information for details) and the most important residues involved are either identical or strongly similar.

To understand the effect of the SH3 domain on the unfolding trajectories, we repeat the same pulling simulations with the two spectrin repeats with the SH3 domain removed. Fig. 2c shows that without the SH3 domain, for both plectin and desmoplakin, the inter-domain interface is the very first feature to be lost during the unfolding pathways. Compared to the previous cases, where the SH3-SR4 interface was usually only lost after SR4 had unfolded, here, the two spectrin repeat domains immediately lose contact and unfold independently. In the case of desmoplakin, there is a clear preference (about 90%): SR4 unfolds before SR5, while for plectin the order is random. We can estimate the difference between the unfolding barriers of the two spectrin repeats, ${\rm{\Delta }}{\rm{\Delta }}{G}_{{\rm{SR5}},S{\rm{R}}4}={\rm{\Delta }}{G}_{{\rm{SR}}5}-{\rm{\Delta }}{G}_{{\rm{SR}}4}$ in desmoplakin from the 3/33 ratio to be most likely ${\rm{\Delta }}{\rm{\Delta }}G=1.2$ kcal/mol (SR4 having a lower barrier than SR5), with the 95% confidence interval between ${\rm{\Delta }}{\rm{\Delta }}G=0.7\ldots 2.0$ kcal/mol (see SI for the Bayesian arguments^42^ in support of this). For plectin, where the preference is 17/33 for SR5, the difference of the barriers has the mode at ${\rm{\Delta }}{\rm{\Delta }}G=-0.04$ kcal/mol, and with the 95% confidence interval between ${\rm{\Delta }}{\rm{\Delta }}G=-0.4\ldots 0.4$ kcal/mol.

### The SH3 insertion stabilizes the plakin domain

The fact that the SH3 domain remains intact until the very last part of the unfolding simulations supports the idea that the insertion has a force-sensing role. In none of the simulations did we observe the SH3 domain to unfold before it loses contact with the spectrin repeats. On the contrary, for a significant part of the unfolding time, it is both fully folded and at the same time its binding site fully solvent-exposed.

**Figure 3:**
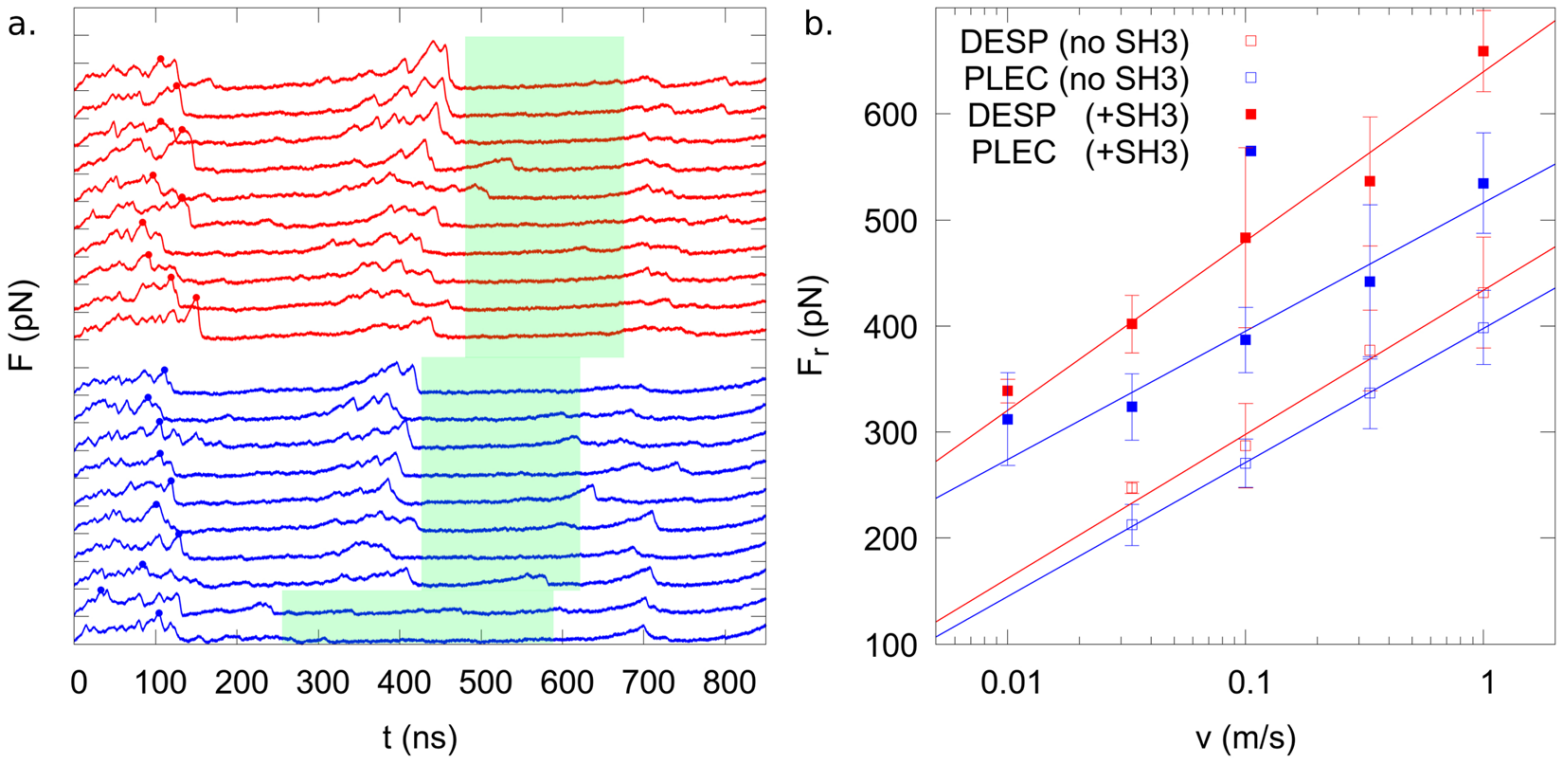
Unfolding forces for desmoplakin (DESP) and plectin (PLEC). (**a**) The force profiles (smoothed with a Gaussian of standard deviation of 1 ns) of desmoplakin (red) and plectin (blue) at the pulling velocity of 0.1 nm/ns. The dots show the highest force within the first peak, i.e., the considered rupture force. We mark with green boxes the region of the pulling trajectories in which the proteins are active (the SH3 domain is freed but fully folded). (**b**) The average rupture forces of desmoplakin and plectin, with and without the SH3 domain, at each pulling velocity. The bars show the standard deviation at each velocity and the line is the Bell model (see Eq. 1) fit of all four considered systems. The distance between the y-ticks is 400 pN.

We next asked whether the SH3 domain also affects the rupture forces of a plakin domain. Fig. 3 summarizes how the rupture force observed for the two proteins depends on the pulling velocity (i.e., the loading rate) and the presence of the SH3 domain. Inserting the SH3 domain into the SRs of both plakin domain constructs increases the rupture force significantly along the whole range of loading rates. The rupture force increase amounts to 48% for desmoplakin and 31% for plectin on average. Clearly, one of the effects of the SH3 domain is the stabilization of this junction.

This consistent drop in rupture forces supports the notion that one main role of this SH3 domain is the mechanical stabilization of the plakin domain. Furthermore, it is noteworthy that the rupture forces for the loss of the two different SH3-SR4 interfaces probed here are significantly higher than any rupture force corresponding to the unfolding of any single domain of the five considered here (SR3-6, SH3). The stabilizing role of the SH3 domain is also corroborated by the fact that all of the simulated unfolding trajectories of the large construct for desmoplakin showed the outer two domains to fully unfold before the unfolding of the central two begins (see Fig. 3a). Thus, the SRs not in contact with the SH3 domain have a lower mechanical resistance than those with a direct interface with the SH3 domain.

### Rupture forces show desmoplakin to be more robust than plectin

We observe that desmoplakin, on average, has rupture forces about 10–20% higher than plectin (Fig. 3). The higher stability of desmoplakin as compared to plectin is significant at the three fastest pulling velocities (where 10 pulling simulations per system have been performed), and is more pronounced when the SH3 domain is present. Interestingly, while the fit with the Bell model (see Eq. 1) is not entirely satisfactory, the observed slope for the four different considered systems is quite consistent: 200–270 pN/(order of magnitude in velocity). This suggests the higher robustness of desmoplakin to also hold at lower pulling velocities such as those probed experimentally.

The similar slopes from the Bell fit can also be interpreted as similar conformational changes at the rate limiting step, supporting the notion of highly resembling unfolding mechanisms of the two plakin domains investigated here. Extrapolating these trends across several orders of magnitude, however, would result in very high unfolding rates at zero force (≈ 1 (μs)^−1^). We refrain here, however, from using more complicated rupture force-loading rate relations to avoid overfitting our data that is clustered along the high loading rate region and only use the fits to compare the stabilities of the four considered systems.

### Alternative models of plectin have no major effect on rupture forces

The higher stability of desmoplakin over plectin is not easily comprehensible given the high structural homology of the two plakin domains. The two major structural differences between the considered desmoplakin and plectin fragments are: (i) a small ruptured helix at the N-terminus of the helix 5B and (ii) a different salt bridge network between the 4A-4B loop and the upstream SH3 loop. To see whether the significant differences in rupture force between desmoplakin can be accounted for by either of these observations, we repeat (at the highest loading rates only) the pulling simulations of plectin on suitably modified models. As Table 1 shows, however, the rupture forces do not significantly change compared to the ones of wild-type plectin. This implies that the details of the structural model have little influence and that the differences in stability of the two proteins have a different source.

**Table 1:** Comparison of rupture forces and standard errors on the mean $\varepsilon$ observed at 10 replicas each pulled at a velocity of 1 m/s for three different plectin models and desmoplakin.

model	F_*r*_ (pN)	*ε* (pN)
desmoplakin	659.5	13.5
plectin 3PE0	534.3	16.6
plectin 3PE0 + 5J1H	526.0	18.3
plectin 3PE0 + mutations	535.8	19.5

### Differences in force profiles correlate with a small cluster of sequence variations

**Figure 4:**
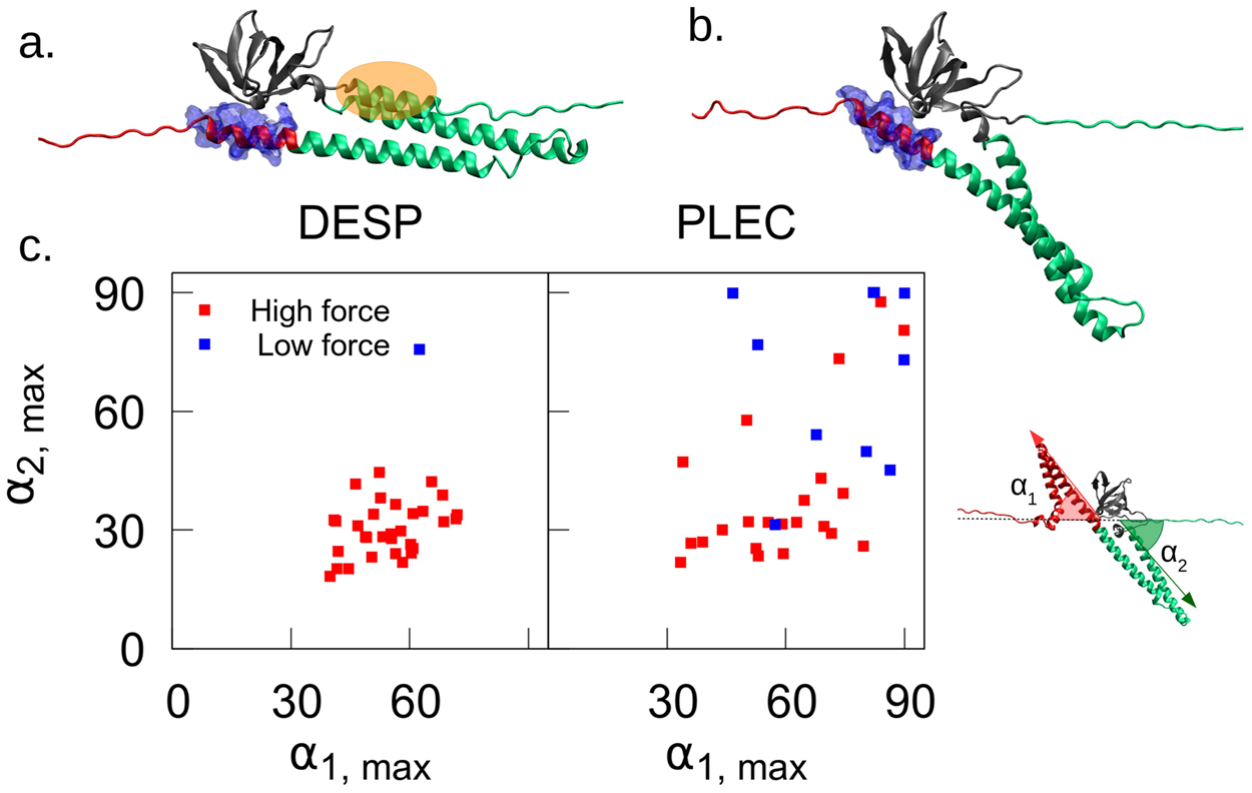
The two different ways the SH3-SR4 interface can be lost: (**a**) “shearing” and (**b**) “tearing” (representative snapshots with red = SR4, green = SR5, blue surface = residues at helix 4 C in contact with the SH3 domain, and orange highlight: the N-terminal domains of helix 5 C, whose unfolding triggers the tilt and therefore the “tearing” topology), and (**c**) the maximum tilt angle observed for the 33 unfolding trajectories of desmoplakin (left) and plectin (right). Red points refer to force profiles with three distinct peaks and blue points show a lack of a middle peak. See also the ROC curve based on this plot in the Supplementary Information.

Paying closer attention to the force profiles of the two considered proteins, we can notice that for desmoplakin, 32/33 of the trajectories feature at least three force peaks, while only 25/33 from plectin do (Fig. 3a). This behavior appears to correlate very closely with a tilting motion of the partially unfolded proteins we observe more frequently for plectin. While in many (or most as for desmoplakin) trajectories the SR helices remain oriented along the pulling direction (Fig. 4a), some unfolding events feature a pronounced tilting of the SRs away from the pulling direction, resulting in a 'tearing' or 'unzipping' of the remaining $\alpha$-helices of the SRs (Fig. 4b). We can quantify this by measuring the maximum angle created by the vectors drawn by (i) the $\alpha$-carbons of N375-P342 (for plectin, these are N858-P825) and the pulling axis and (ii) N375-Y403 (for plectin, N858-Y886) and the pulling axis (Fig. 4c). We denote these tilting angles ${\alpha }_{1}$ and ${\alpha }_{2}$, respectively. Fig. 4c shows that the magnitude of the maximum tilting very well predicts whether an unfolding happens at a high or low force in the second peak. In fact, the highest of the two maximum tilting angles has a receiver-operating characteristic of the high/low force of rupture events with an area under curve of 0.919 (see Supplementary Information for details). More tilting leads to unfolding at lower forces since tilting exposes the SH3-SR4 interface directly to the external force. Tilting also allows the gradual unzipping of SRs from as opposed to a shearing mechanism which requires higher forces, much like in the case of the $\beta$-barrel of GFP^43^.

The higher prevalence of the tilting for plectin rather than desmoplakin can be rationalized by a small patch of sequence variations at the N-terminal of the helix 5 C (the orange patch in Fig. 4a). For plectin, this helix unfolds more often and allows the tilting to occur (since the helix loses contact to helices 5 A and B), while for desmoplakin, even in the cases where helix 5 C loses its secondary structure, it retains contacts with the rest of the spectrin repeat. As a consequence, the SH3 domain of plectin is activated with significantly less work. In addition, plectin unfolding and activation requires a lower rupture force in general (Fig. 3b), independent of the described tilting, further stressing its lower resilience against the external pulling force.

**Figure 5:**
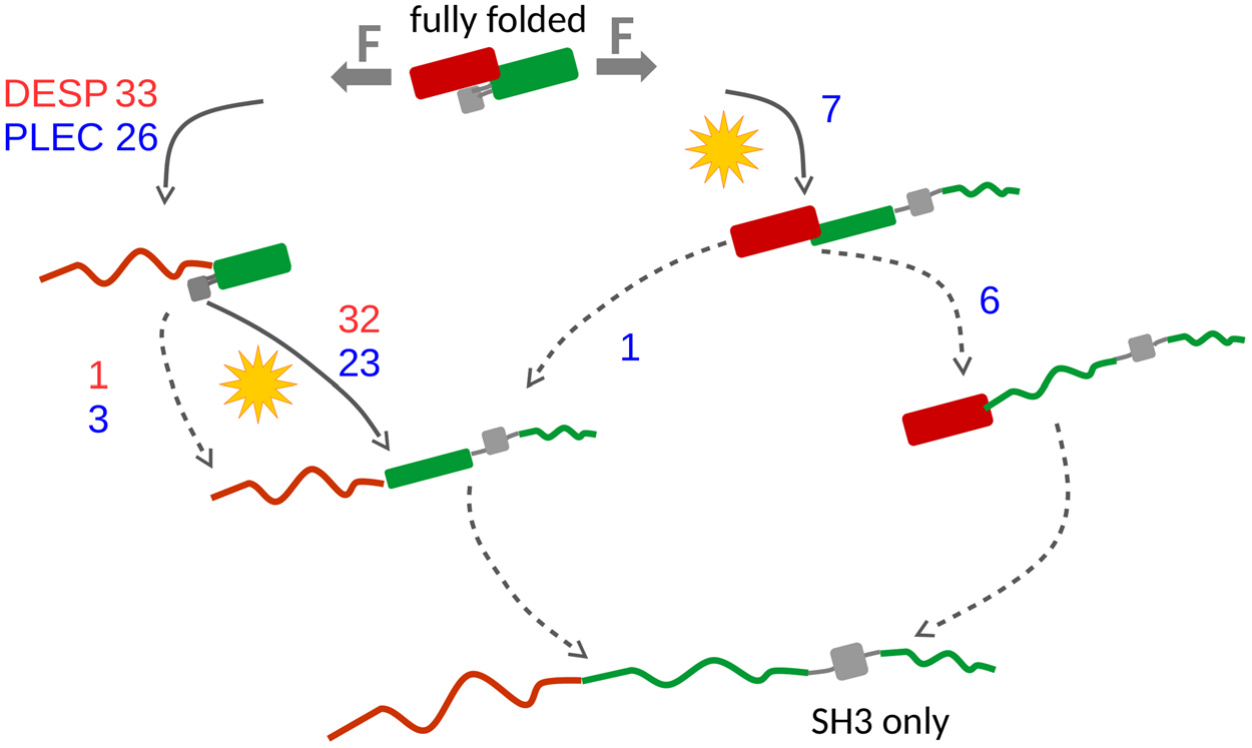
The various unfolding mechanisms exhibited by desmoplakin (red numbers) and plectin (blue numbers). The SH3 domain is represented as a gray box, while SR4 and SR5 are shown in red and green, respectively. Solid arrows show force-resistant unfolding events, while dashed arrows force-compliant ones and an orange star represents the rupture event that activates the SH3 domain. Plectin samples a more diverse set of unfolding sequences, including about a quarter of them with an early activation of the SH3 domain followed by a forceless unfolding.

A summary of the various unfolding possibilities is given in Fig. 5. It is easy to see that plectin exhibits much more variation in unfolding pathways and that all force-resistance of the protein is lost when the SH3-SR4 interface is lost.

## Conclusions

The location of an SH3 domain, an adaptor protein, in the middle of spectrin repeats, known as force-buffering structural elements is striking the eye. Remarkably, it is a conserved feature of the plakin family as well as $\alpha$-spectrin. What is the evolutionary advantage of inserting a highly conserved and widespread protein-protein interaction domain, even more so in its auto-inhibited conformation, right into spectrin repeats, which elastically deform under tension? To address this question, we here analyzed the force response of the central plakin domain of two representatives of the plakin family, namely, desmoplakin and plectin. For both proteins, we identified a likely mechano-sensory role of the SH3 insertion: under force, this domain is invariably freed and can thereafter interact with binding partners (Fig. 5). We have not observed any trajectories in which the domain even partially unfolds before it is released from the SH3-SR interface.

For plectin, in fact, there is some experimental evidence that shows that the SH3 domain is occluded under normal circumstances but binds when free: a recent study^20^, using co-immunoprecipitation, showed that when a construct similar to our considered system (exons 16–24 roughly form SR4-6) interacts with the protein MAP1, it has less affinity to it than when the SH3 domain alone is interacting. For desmoplakin, on the other hand, no SH3 binding partners have been identified, but our data speaks, by similarity to plectin, for a binding-ready SH3 domain of desmoplakin in tensed desmosomes. We speculate that desmoplakin’s SH3 domain by itself, or with adjacent but unfolded spectrin repeat fragments, might be the protein unit interacting favorably with substrates, and could be the construct of choice when searching for binding partners.

The two proteins investigated in this work (desmoplakin and plectin) exhibited similar unfolding scenarios across two orders of magnitude of pulling velocities. Given that the sequence identity between the two proteins of 42% is comparable to the sequence identities within the family (23–64%, see SI), we speculate that all plakin family members^44^ as well as the related $\alpha$-spectrin follow a highly similar picture of SH3 mechano-sensing. However, there are also some significant differences between the two proteins. In particular, in the case of plectin, the SH3 domain can lose contact with the rest of the plakin domain with relatively reduced resistance due to a different topology of pulling characterized by a net tilting of the entire protein structure. We attribute this change in behavior to a relatively small pocket of nonconservative sequence variations at the beginning of the 5C $\alpha$-helix.

Extrapolating our conclusions to the physiological case is quite difficult, given how dense desmosomes or hemidesmosomes are. In particular, the length scale of the full unfolding in our simulations (about 100–150 nm) seems very large, yet should not be *per se* excluded to occur. Also, the interactions with neighboring structures could tether the protein in such a way as to concentrate the stress on this particular interface. Similarly, the levels of force acting on single desmoplakin or plectin proteins in the cell remain to be quantified such as with in-cell force sensors^45^. It is interesting to note that force-induced SH3 activation in plakin domains as found here requires force levels similar to those needed for talin activation^46^, a reminiscent force-sensing mechanism of focal adhesion sites^47^. Furthermore, a wide range of proteins (including the structurally similar alpha-spectrin) are known to mechanically unfold when cells are stretched^48^.

There is strong evidence that the plakin domain and in particular the SR-SH3 interface play a key role in the stability or function of the proteins, therefore under stress in physiological conditions. For desmoplakin, 16 out of the known 25 disease mutations are in the central plakin domain^21^ (9 of which are in the simulated fragment SR4-5 - see Fig. S11 for their location). Despite these difficulties, the order of unfolding observed here is valuable knowledge as these insertions are observed in other proteins as well.

Direct validation with force spectroscopy experiments, preferably including ones with very high loading rates, would further help understanding the force response of these proteins. These and other future efforts will shed further light onto the role of the SH3 domain insertion in tissue integrity and stress response.

## Electronic supplementary material

Supplementary Video 1

Supplementary Video 2

Supplementary Information
