# Supplementary material for: The mechano-sensing role of the unique SH3 insertion in plakin domains revealed by Molecular Dynamics simulations
Source: Sci Rep. 2017 Sep 15;7:11669. doi: 10.1038/s41598-017-11017-2 (PMC5601466; doi:10.1038/s41598-017-11017-2)
Supplement: Supplementary file 3 — Supplementary Information [file 41598_2017_11017_MOESM3_ESM.pdf]

Supporting information for: The mechano-sensing role of the unique SH3 insertion in plakin domains revealed by Molecular Dynamics simulations

Csaba Daday[1, 2], Katra Kolšek[2], and Frauke Gräter[1, 2, *]

[1]Interdisciplinary Center for Scientific Computing (IWR), Heidelberg University, Mathematikon, INF 205, 69120 Heidelberg, Germany
[2]Heidelberg Institute for Theoretical Studies, Schloß-Wolfsbrunnenweg 35, 69118 Heidelberg, Germany
[*]frauke.graeter@h-its.org

August 1, 2017

Constructing alternative models for plectin

We attempted to construct a model plectin closer to desmoplakin in two different ways.

Removing helix B0

There is a 10-residue long helix B0 before the N-terminus of helix 5B in the PDB structure 3PE0 plectin, which is not observed in desmoplakin, and is similarly not observed in a group of new crystal structures, for instance 5J1H. Since this new crystal structure does not contain the SH3 domain but only a five-residue linker, we model the B0-less plectin as a homology model with both templates.

Introducing additional salt bridges

We introduce the following five mutations (according to the amino acid numbering of plectin): R784K, A937D, P973R, H975K, E1001L (shown in boldface in Figure S1). These are introduced to mimic the salt-bridge network of desmoplakin. The following salt bridges are possible after these mutations: D780-R973, D783-K932, D937-K784, and E773-K975 (see Figure S3). E1001L was

introduced to hinder a salt bridge forming with K975 (observed in the wild-type simulations), which would not strengthen the interface with SR4.

We simulate this mutated structure for 1 μs and choose 10 frames in which at least two salt bridges are observed, although this happens in only a minority of the equilibrium trajectory. While these chosen form a biased set of frames, this only strengthens the negative findings of the main text (that the additional salt bridges do not increase the required rupture forces of unfolding).

Longest-lived interactions at the SH3-4C5A interface

We list the ten SH3 domain-helix 4C5A residue pairs that have the longest interaction lifetime. We define an interaction between a residue pair if at least two heavy atoms are within 6Å from each other. We define the average interaction "lifetime" as $x_{ij} = \frac{1}{33}\sum_{a=1..33} v_a t_a^{ij}$, where a is the index of each unfolding trajectory and t_a^{ij} is the total time of interaction between residues i and j. We do not exclude non-native contacts from the analysis, but the residue pairs in the following list are all native contacts.

Tables S2 lists the identified residue pairs and residues for desmoplakin and plectin. 7 out of 10 residue pairs are common in desmoplakin and plectin. Furthermore, the hydrogen bond between the backbone of V508 (V991 for plectin) the SH3 domain and the side chain of H371 (H854 for plectin) in the helix is the longest lived for both proteins. The four residues in the SH3 domain and the four residues in helix 4C5A that form these 7 residue pairs are shown in Figure S4. As Table S2 shows, the respective residues in the two proteins are strongly similar for three and identical for five positions in the alignment.

The interactions at the desmoplakin interface are longer lived out by approximately 7-8 nm of average extension. About 4 nm out of this can be explained by the different number of residues in our models (274 for plectin, 287 for desmoplakin), and the other 3-4 nm is due to the two different unfolding pathways exhibited by plectin.

As Figures S5 and S6 show, these interactions break at very similar times across all 33 trajectories. The major difference between them is that several of these interactions at the interface are disturbed even in the "fully folded" state. The minority of about 8 trajectories in plectin where the SH3 domain is activated in the first rupture event (around 12-15 nm of extension) is also clearly discernible.

Estimating the difference in barriers for the two spectrin repeat unfoldings

If we assume that the unfolding of the two spectrin repeats is independent from each other, the ratio r_{SR4} of unfolding simulations in which SR4 is observed to

$\text{res}_i^{\text{SH3}}$	$\text{res}_j^{\text{4C5A}}$	i_{al}	j_{al}	x_{ij} (nm)
**Desmoplakin**				
V508	H371	**240**	**103**	38.5
L466	H371	**198**	**103**	38.1
L466	V370	198	102	37.9
V508	I368	**240**	**100**	37.6
I511	H371	**243**	**103**	37.4
L510	H371	**242**	**103**	37.3
S507	H371	239	103	37.2
G509	H371	241	103	37.1
I511	E374	**243**	**106**	36.7
V508	C367	**240**	**99**	36.4
**Plectin**				
$\text{res}_i^{\text{SH3}}$	$\text{res}_j^{\text{4C5A}}$	i_{al}	j_{al}	x_{ij} (nm)
V991	H854	**240**	**103**	31.1
L994	E857	**243**	**106**	30.6
V949	H854	**198**	**103**	30.0
V991	C850	**240**	**99**	29.6
C950	C850	199	99	29.5
L994	H854	**243**	**103**	29.3
V991	I851	**240**	**100**	28.5
V949	C850	198	99	28.3
V991	L847	240	96	28.2
F993	H854	**242**	**103**	27.9

Table S1: The top 10 longest-lived contacts in desmoplakin and plectin, averaged across all 33 unfolding trajectories. i_{al} and j_{al} refer to indices in the alignment starting from the N-terminus of the crystal structure 3PE0 (L752 for plectin, L269 for desmoplakin). In bold face: aligned indices of pairs that appear in the top 10 list of both proteins.

i_{al}	Desmoplakin	Plectin	Sim.
**SH3 domain**			
198	L466	V949	:
240	V508	V991	$*$
242	L510	F993	:
243	I511	L994	:
**Helix 4C5A**			
j_{al}	Desmoplakin	Plectin	Sim.
99	C367	C850	$*$
100	I368	I851	$*$
103	H371	H854	$*$
106	E374	E857	$*$

Table S2: The residues that appear in pairs of both top 10 lists and their similarity ($*$ and : refer to identity and strong similarity, respectively).

unfold first is given by

$$r_{\mathrm{SR4}} = \frac{e^{-\Delta g_4}}{e^{-\Delta g_4} + e^{-\Delta g_5}} = \frac{1}{1 + e^{\Delta g_4 - \Delta g_5}} = \frac{1}{1 + e^{-\Delta\Delta g}}, \tag{1}$$

where $\Delta g_i = \frac{\Delta G_i}{kT}$ and $\Delta\Delta g = \Delta g_5 - \Delta g_4$, where ΔG_i is the barrier of unfolding of one of the spectrin repeats (see Fig. S7).

We can reasonably assume a lack of loading-rate dependence of the systems, in which case the division of the 33 unfolding experiments will follow a binomial distribution with success rate r_{SR4}. The conjugate prior for a binomial distribution is the beta distribution, and we will start from a flat prior, $p(r) = \beta(1,1)(r) = 1, \forall r \in [0,1]$. The final probability distribution for the success rate will simply be given by:

$$p(r) = \beta(N_4 + 1, N_5 + 1)(r) \propto r^{N_4} \cdot (1 - r)^{N_5}, \tag{2}$$

where N_4 and N_5 show the number of simulations with first unfoldings of SR4, SR5, respectively. We plot the probabilities for the "true ratios" for desmoplakin ($N_4 = 30$, $N_5 = 3$) and plectin ($N_4 = 16$, $N_5 = 17$) in Figure S8a.

To obtain the probability distribution of the difference in barriers, we need to keep into account the derivative of the ratio with respect to it, and obtain:

$$p(\Delta\Delta g) \propto \beta(N_4 + 2, N_5 + 2)(r(\Delta\Delta g)) \propto r^{N_4+1} \cdot (1 - r)^{N_5+1}, \tag{3}$$

given that $|dr/d\Delta g| = \frac{e^{\Delta g}}{(1+e^{\Delta g})^2} = r(1-r)$. The final distribution of the difference of unfolding energies is in Figure S8b. The modes and 95% confidence intervals reported in the main text are based on this distribution.

Quantifying the connection between low-force unfolding and tilting

We can quantify the predictive power of tilting of a lack of a second peak in the force profile by computing the area under a reciever operating curve (ROC). If we consider the predictor the angle $\alpha_{\max} = \max(\alpha_{1,\max}, \alpha_{1,\max})$ and low force any unfolding trajectory with a second peak of at most 150 kJ/mol/nm (as in Fig 4c in the main text), we obtain an excellent result of AUC=0.919, indicative of excellent predictive power (see Fig S9 for the raw data).

On the use of a 5 fs time step and virtual sites

In the main text, we employed a 5 fs time step, virtual sites, and freezing all bonds. We compare here this protocol to the more common procedure of using a 2 fs time step and freezing only hydrogen-heavy atom bonds (with the same force field). To this end, we simulated the largest construct, namely, the SR3-6 fragment from desmoplakin. We performed an equilibrium simulation of 200 ns and three pulling simulations of 140 ns each at the highest pulling velocity, 1

m/s. Fig S10 shows that the unfolding trajectories are fully independent of the time step.

Interestingly, the rupture forces observed with a 2 fs time step are about 19% lower than those with a 5 fs time step. With a time step of 2 fs, we observe rupture forces between 504 and 616 pN, with a mean at 550 pN. The similar rupture forces with 5 fs was between 614..786 pN with a mean at 681 pN. In this case, we consider the rupture force the highest force observed between event 60..80 nm, i.e., the event we considered also as the rupture force in the main text on the smaller construct.

```
                         10        20        30        40        50        60
                          |         |         |         |         |         |
DESP_HUMAN       LRQLQNIIQATSREIMWINDCEEEELLYDWSDKNTNIAQ-KQEAFSIRMSQLEVKEKELN
PLEC_HUMAN       LESLHSFVAAATKELMWLNEKEEEEVGFDWSDRNTNMTA-KKESYSALMRELELKEKKIK
MACF1_HUMAN      LQSLHKFVSRATAELIWLNEKEEEELAYDWSDNNSNISA-KRNYFSELTMELEEKQDVFR
DYST_HUMAN       LDTLHNFVSRATNELIWLNEKEEEEVAYDWSERNTNIAR-KKDYHAELMRELDQKEENIK
EVPL_HUMAN       LGSLYTHLQGCTRQLSALAEQQRRILQQDWSDLMADPAGVRREYEHFKQHELLSQEQSVN
PEPL_HUMAN       LSSLQDYMQRCTNELYWLDQQAKGRMQYDWSDRNLDYPSRRRQYENFINRNLEAKEERIN
                 *  *   :   : ::  : :  .  :  ***:   : .  :::       :*  ::. ..

                         70        80        90       100       110       120
                          |         |         |         |         |         |
DESP_HUMAN       KLKQESDQLVLNQHPASDKIEAYMDTLQTQWSWILQITKCIDVHLKENAAYFQFFEEAQS
PLEC_HUMAN       ELQNAGDRLLREDHPARPTVESFQAALQTQWSWMLQLCCCIEAHLKENAAYFQFFSDVRE
MACF1_HUMAN      SLQDTAELLSLENHPAKQTVEAYSAAVQSQLQWMKQLCLCVEQHVKENTAYFQFFSDARE
DYST_HUMAN       SVQEIAEQLLLENHPARLTIEAYRAAMQTQWSWILQLCQCVEQHIKENTAYFEFFNDAKE
EVPL_HUMAN       QLEDDGERMVELRHPAVGPIQAHQEALKMEWQNFLNLCICQETQLQHVEDYRRFQEEADS
PEPL_HUMAN       KLHSEGDQLLAAEHPGRNSIEAHMEAVHADWKEYLNLLICEESHLKYMEDYHQFHEDVKD
                 .:.. .: :    **.   :::.  ::: : .   ::  * : :::    * .* .:. .

                        130       140       150       160       170       180
                          |         |         |         |         |         |
DESP_HUMAN       TEAYLKGLQDSIRKKYPCDKNMPLQHLLEQIKELEKEREKILEYKRQVQNLVNKSKKIVQ
PLEC_HUMAN       AEGQLQKLQEALRRKYSCDRSATVTRLEDLLQDAQDEKEQLNEYKGHLSGLAKRAKAVVQ
MACF1_HUMAN      LESFLRNLQDSIKRKYSCDHNTSLSRLEDLLQDSMDEKEQLIQSKSSVASLVGRSKTIVQ
DYST_HUMAN       ATDYLRNLKDAIQRKYSCDRSSSIHKLEDLVQESMEEKEELLQYKSTIANLMGKAKTIIQ
EVPL_HUMAN       VSQTLAKLNSNLDAKYSPAPGGPPGAPTELLQQLEAEEKRLAVTERATGDLQRRSRDVAP
PEPL_HUMAN       AQELLRKVDSDLNQKYGPDFKDR-YQIELLLRELDDQEKVLDKYEDVVQGLQKRGQQVVP
                     *  :.. :  **              :::   :.: :   :    .*  :.: :

                        190       200       210       220       230       240
                          |         |         |         |         |         |
DESP_HUMAN       LKPRNPDYRSNKPIILRALCDYKQDQKIVHKGDECILKDNNERSKWYVTGPGGVDMLVPS
PLEC_HUMAN       LKPRHPAHPMRGRLPLLAVCDYKQVEVTVHKGDECQLVGPAQPSHWKVLSSSGSEAAVPS
MACF1_HUMAN      LKPRSPDHVLKNTISVKAVCDYRQIEITICKNDECVLEDNSQRTKWKVISPTGNEAMVPS
DYST_HUMAN       LKPRNSDCPLKTSIPIKAICDYRQIEITIYKDDECVLANNSHRAKWKVISPTGNEAMVPS
EVPL_HUMAN       LPQRRN--PPQQPLHVDSICDWDSGEVQLLQGERYKLVDNTDPHAWVVQGPGGETKRAPA
PEPL_HUMAN       LKYRRE--TPLKPIPVEALCDFEGEQGLISRGYSYTLQKNNG-ESWELMDSAGNKLIAPA
                 *  *         : : ::**:   :  : :.    *        * : .. *    .*:

                        250       260       270
                          |         |         |
DESP_HUMAN       VGLIIPPPNPLAVDLSCKIEQYYEAILALWNQLYI
PLEC_HUMAN       VCFLVPPPNQEAQEAVTRLEAQHQALVTLWHQLHV
MACF1_HUMAN      VCFLIPPPNKDAIEMASRVEQSYQKVMALWHQLHV
DYST_HUMAN       VCFTVPPPNKEAVDLANRIEQQYQNVLTLWHESHI
EVPL_HUMAN       ACFCIPAPDPDAVARASRLASELQALKQKLATVQ-
PEPL_HUMAN       VCFVIPPTDPEALALADSLGSQYRSVRQKAAGSK-
                 . : :*..:  *      :    . :
```

Figure S1: Alignment between desmoplakin (DESP, accession number P15924), plectin (PLEC, accession number Q15149), microtubuli-actin crosslinking factor 1 (MACF1, accession number Q9UPN3), dystonin (DYST accession number Q03001), evoplakin (EVPL, accession number Q92817) and periplakin (PEPL, accession number O60437), when considering only the 274 residues in the PDB 3PE0. In boldface: the five mutations produced in the desmoplakin-plectin hybrid model. The last line shows identity (∗) and strong (:) or weak (.) similarity. The alignment was performed using ClustalW, version 1.8, with default parameters. In orange: the patch of 6/8 non-conserved residues at the N-terminus of helix 5C. In blue and green: the residues in the SH3 and helix 4C5A with the longest-lived interactions along the unfolding trajectory (see Table S2).

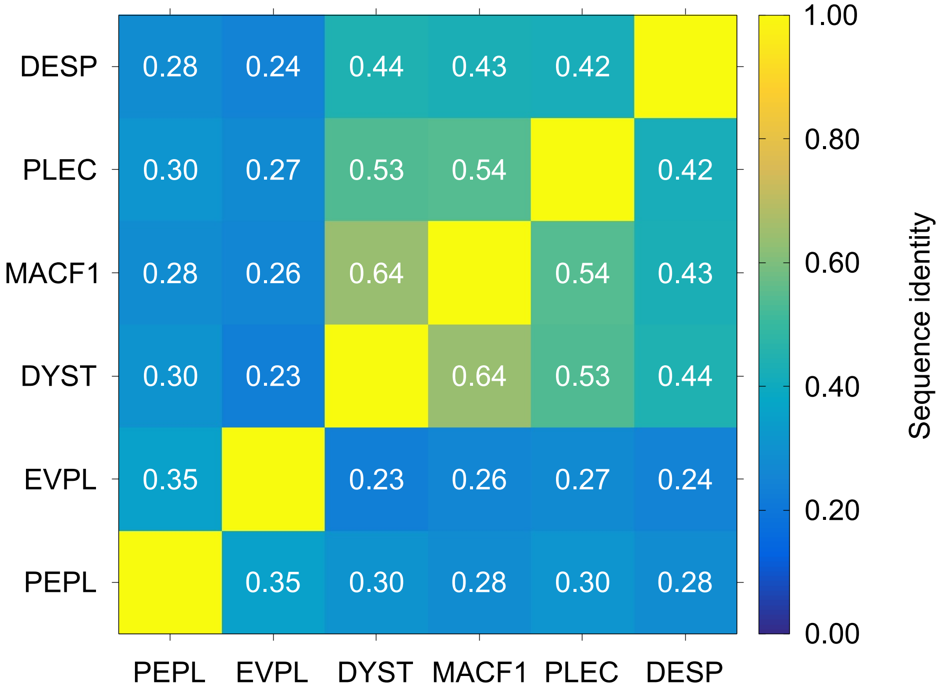

Figure S2: Mutual sequence identity between the six proteins (based on the alignment in the previous figure).

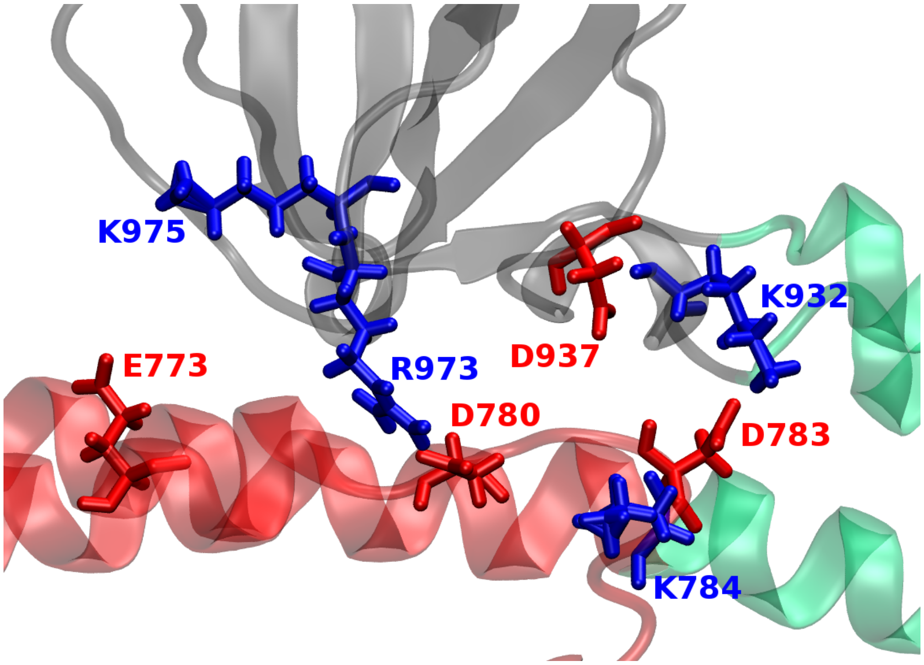

Figure S3: The position of the four possible salt bridges in the 5-mutant in plectin: D780-R973, D783-K932, D937-K784, and E773-K975.

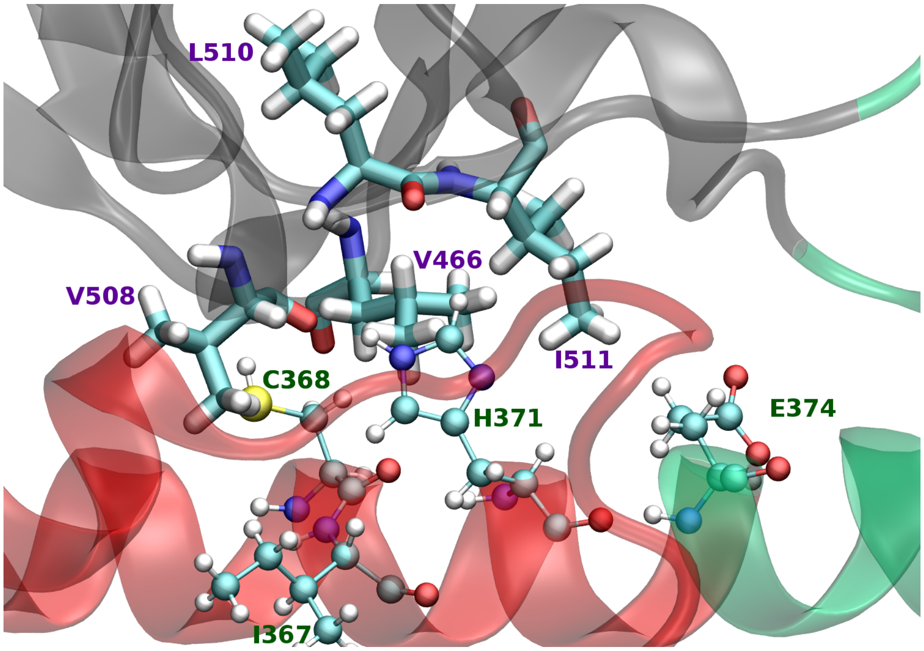

Figure S4: The four residues in the SH3 domain (licorice, purple labels) and the four residues in the 4C5A helix that form the longest-lived 7 interfracial contacts in desmoplakin and plectin. We present the residues on desmoplakin.

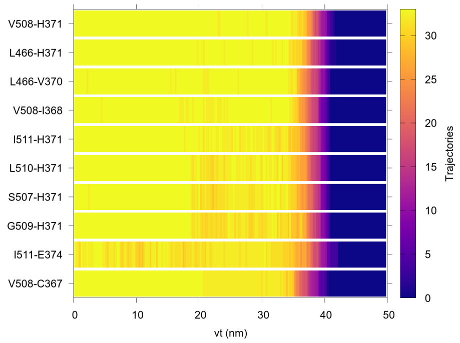

Figure S5: The lifetime of the most long-lived interactions at the SH3-4C5A interface for desmoplakin. The x-axis shows the inter-spring distance relative to the initial positions, while the color scheme counts how many trajectories out of the 33 still contain this interaction.

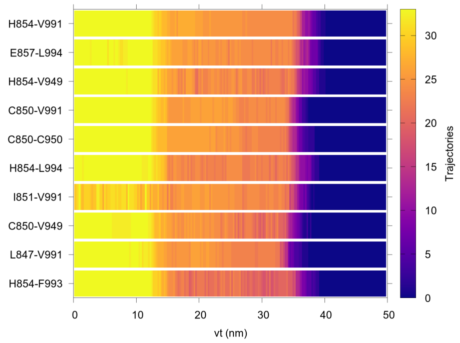

Figure S6: The lifetime of the most long-lived interactions at the SH3-4C5A interface for plectin. The x-axis shows the inter-spring distance relative to the initial positions, while the color scheme counts how many trajectories out of the 33 still contain this interaction.

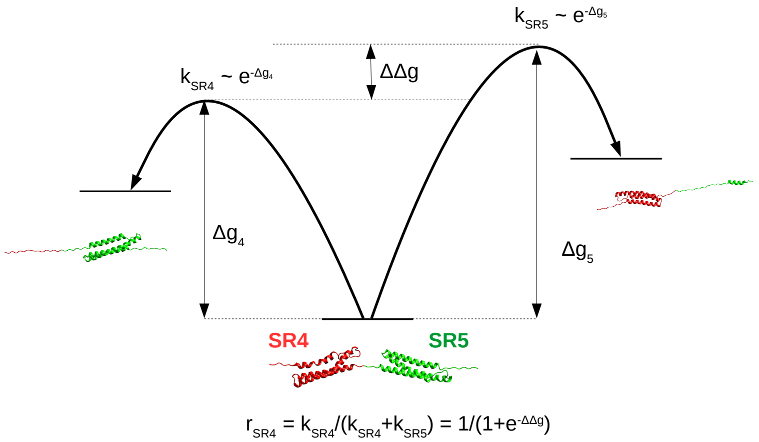

Figure S7: The two different paths the SR4-SR5 (with the SH3 domain removed) unfolding can go through. We denote the rates with $k_{SR4,5}$ and the probability of SR4 unfolding first with r_{SR4}.

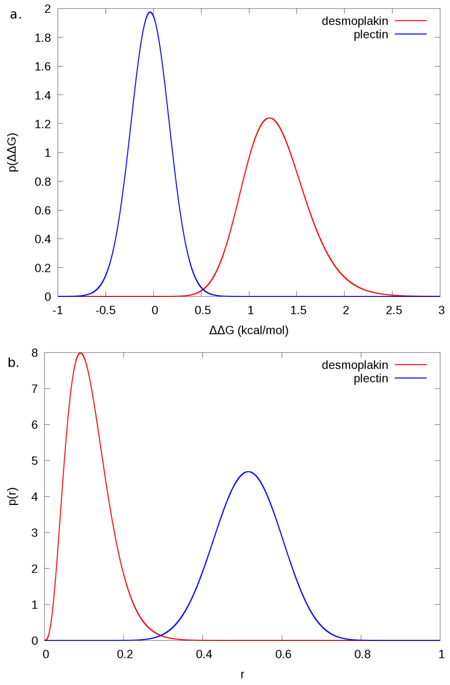

Figure S8: a: Probability distribution for the difference between the barriers of SR4 and SR5 (in kcal/mol) based on the observed ratio of unfolding simulations. b: Probability distribution for the "true ratio" of first unfolding of SR4 based on the observed ratio.

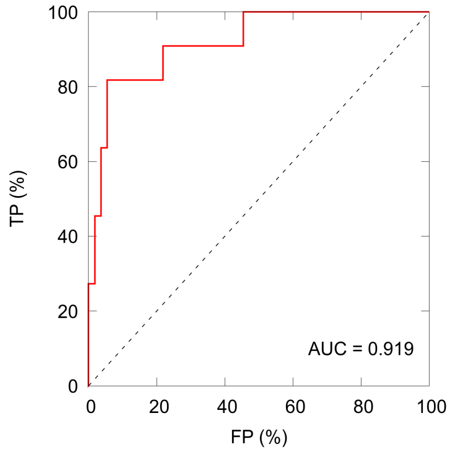

Figure S9: Receiver operating characteristic showing the suitability of the maximum tilting angle to predict a low-force second unfolding event. TP stands for true positives (low force with high angle) and FP for false positives (high force with high angle). All 66 unfolding trajectories of both desmoplakin and plectin were used, 11 of them with low-force and 55 with high-force second peaks. The area under curve (AUC) of 0.919 shows that this maximum tilting angle is an excellent predictor of a low-force second peak.

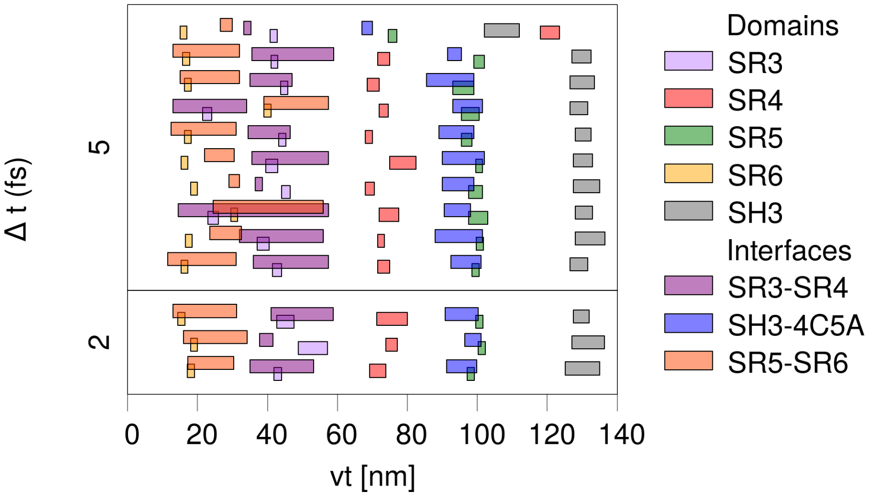

Figure S10: Unfolding events of the SR3-6 system simulated with 2 fs vs. 5 fs time steps.

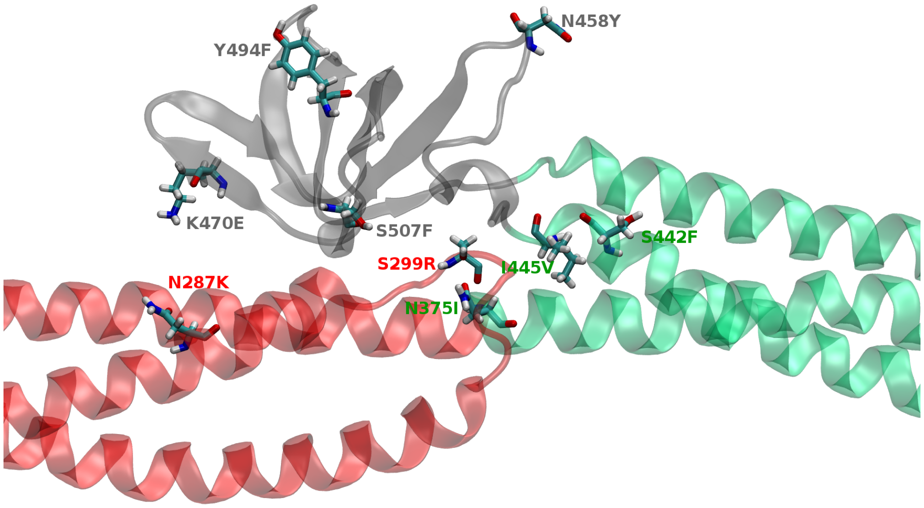

Figure S11: The location of the 9 arrythmogenic right ventricular cardiomyopathy (ARVC)-related mutations that are found in the main simulated segment.
